# Divergence in Glyphosate Susceptibility between *Steinchisma laxum* Populations Involves a Pro106Ser Mutation

**DOI:** 10.3390/plants12183315

**Published:** 2023-09-20

**Authors:** Veronica Hoyos, Guido Plaza, Candelario Palma-Bautista, Jose G. Vázquez-García, José Alfredo Dominguez-Valenzuela, Ricardo Alcántara-de la Cruz, Rafael De Prado

**Affiliations:** 1 Departamento de Ciencias Biológicas, Universidad Nacional de Colombia, Palmira 763533, Colombia; 2 Departamento de Agronomía, Universidad Nacional de Colombia, Bogotá 111321, Colombia; 3 Departamento de Parasitología Agrícola, Universidad Autónoma Chapingo, Texcoco 56230, Mexico; 4 Agroforestry and Plant Biochemistry, Proteomics and Systems Biology, Department of Biochemistry and Molecular Biology, University of Cordoba, 14014 Cordoba, Spain; 5 Departamento de Química, Universidade Federal de São Carlos, São Carlos 13565-905, Brazil

**Keywords:** *EPSPS* gene, lax panicgrass, N-(phosphonomethyl) glycine, TSR mechanisms, rice cultivation

## Abstract

The characterization of the mechanisms conferring resistance to herbicides in weeds is essential for developing effective management programs. This study was focused on characterizing the resistance level and the main mechanisms that confer resistance to glyphosate in a resistant (R) *Steinchisma laxum* population collected in a Colombian rice field in 2020. The R population exhibited 11.2 times higher resistance compared to a susceptible (S) population. Non-target site resistance (NTSR) mechanisms that reduced absorption and impaired translocation and glyphosate metabolism were not involved in the resistance to glyphosate in the R population. Evaluating the target site resistance mechanisms by means of enzymatic activity assays and *EPSPS* (5-enolpyruvylshikimate-3-phosphate synthase) gene sequencing, the mutation Pro106Ser was found in R plants of *S. laxum*. These findings are crucial for managing the spread of *S. laxum* resistance in Colombia. To effectively control *S. laxum* in the future, it is imperative that farmers use herbicides with different mechanisms of action in addition to glyphosate and adopt Integrate Management Programs to control weeds in rice fields of the central valleys of Colombia.

## 1. Introduction

Almost 50% of the world population consumes rice as an important component of their diet [1]. In Colombia, rice cultivation has a crucial role in ensuring food security, rural development, environmental sustainability, foreign trade, and supply sufficiency [2]. Ranking 47 out of 176 countries, Colombia’s per capita consumption of white rice is 42.9 kg, close to the global average of 42.5 kg [3], highlighting the importance of this crop in the country. However, weed management poses a constant challenge for rice growers, leading to significant losses in yield and quality [4]. Moreover, herbicide resistance is becoming a major challenge in rice production across the Americas [5,6].

The occurrence of herbicide resistance represents a complex evolutionary and ecological phenomenon, including two principles factors: management (frequency, herbicide rate, chemistry of herbicides used, and alternative weed controls) and weed species (environmental and biological characteristics of the species and molecular and biochemical mechanisms capable of conferring resistance) [7]. The emergence of resistant weed populations leads to different agronomic consequences in production systems, such as yield losses, decreased control options, increased use of herbicides due to additional applications or dose increases, increased rates of herbicides, and production costs [8].

**Figure 1:**
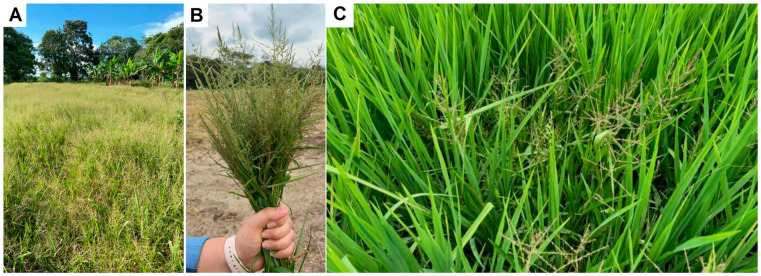
(**A**,**C**) Commercial rice field infested by glyphosate-resistant *S. laxum* in the central valleys of Colombia. (**B**) Prospecting and identification of *S. laxum* by a Fedearroz technician.

In Colombia, several cases of resistance to herbicides have been reported in recent years [9,10], including the first case of glyphosate resistance in *Chloris radiata* [11], a species that was classified as a troublesome weed for rice in the central zone of Colombia since 2011 [10]. A similar situation has occurred with *Steinchisma laxum* (Sw.) Zuloaga (synonymy: *Panicum laxum* Sw.), a weed that had no economic importance in the country, but in recent years has become a problem requiring management. *Steinchisma laxum*, commonly known as lax panicgrass, is a C3 native species present in Colombia, belonging to the Panicoideae subfamily, growing at altitudes ranging from 500 to 1500 m [12], in open flooded savannahs, humid savannahs, and marsh lands [13]. In investigations carried out by the National Federation of Rice Growers of Colombia (Fedearroz), this species is commented as emergent in production lots in different regions of the country, currently infesting around 20 to 30% of the area of each lot and present in more than 50% of the fields in the department of Meta (Bautista, F. personal communication) (Figure 1).

Identifying and alerting the occurrence of resistance to herbicides is a tool that helps the different levels of the agricultural production chain to formulate efficient and appropriate strategies to control weeds in the field [14]. To contribute to solving this problem, it is necessary to have a deep understanding of the biology, physiology, genetics, epigenetics, ecology, population dynamics, and the dispersal mechanisms of resistant weeds [15]. For *S. laxum*, there is no information on the biology, ecology, and in situ management, for which the control is carried out with products such as glyphosate (N-(phosphonomethyl) glycine), which is included as the first alternative because of its broad spectrum activity and its low cost [16]. Glyphosate works by inhibiting the enzyme 5-enolpyruvylshikimate-3-phosphate synthase (EPSPS) within the shikimic acid metabolic pathway [17]. This inhibition leads to the disruption of synthesis of aromatic amino acids, synthesis of flavonoids, lignin formation, monolignol polymerization, and the production of other phenolic compounds, as well as the disruption of carbon flow through other essential metabolic pathways [17,18].

In rice cultivation, the stages that justify these herbicides are pre-sowing treatments to condition the field or after the harvest, i.e., during the initiation of soil preparation work [19]. This high use of glyphosate in rice cultivation in Colombia has exerted strong selection pressure on both established and emerging weed species, leading to the development of resistance to this herbicide [9,10]. The objectives of this research include the following: (1) to assess the glyphosate susceptibility level in two populations of *S. laxum* and (2) to elucidate the mechanisms of both non-target and target site types to gain insights into the resistance of this weed.

## 2. Materials and Methods

### 2.1. Biological Material

Multiple suspected glyphosate-resistant populations of *S. laxum* were gathered in commercial rice fields from the central zone and Llanos plains of Colombia to perform a screening test with the recommended field rate of the herbicide (1080 g ae ha^−1^). Ripe panicles were removed from adult plants in the flowering stage, stored in paper bags, labeled, and dried at 40 °C for 4 days. Once dry, the seeds were detached from the panicle, and a seed pool of at least 25 plants was created for each population. In this study, the mechanisms of glyphosate resistance were characterized in the population with the highest resistance level in comparison with a susceptible (S) population gathered from a livestock lot without the use of any herbicides (San Martín [Meta], 3.535658°, −73.423100°). Thus, seeds from the resistant (R) population were gathered in Meta (4.0282611°, −73.4758694°).

The seeds of R and S populations were planted in trays containing peat, which was appropriately moistened, and then covered with plastic film. The trays were positioned inside a growth chamber at 28/18 °C (day/night), featuring a 12 h photoperiod, a light density of 350 μmol m^−2^ s^−1^, and 60% relative humidity. Trays were kept there until the seedlings developed two or three true leaves (18–20 days after sowing). The seedlings were then transplanted into plastic pots measuring 10 × 10 cm × 6.3 cm, containing a soil/peat mixture at a ratio of 4:1 (*v*/*v*). Throughout the experiments, pots were maintained in a greenhouse under controlled conditions (12 h photoperiod, 32/23 °C day/night, and 60–70% relative humidity), mirroring the typical rice growth conditions in Colombia [10,20]. Plants were watered as required to uphold the substrate’s field capacity.

### 2.2. Glyphosate Dose–Response Experiments

To assess the differences in the level of susceptibility to glyphosate, both S and R *S. laxum* populations were subjected to dose–response assays of glyphosate (Roundup^®^ SL, 36% *w*/*v*, Bayer CropScience, Leverkusen, Germany). Plants were treated at the tillering stage (4–6 true leaves), and the doses tested for the S population were 0, 50, 100, 200, 300, 600, 800, and 1000 g ae ha^−1^, while for the R population, the doses were 0, 1000, 2000, 3000, 4000, 6000, 8000, and 10,000 g ae ha^−1^. Herbicide treatments were conducted in a laboratory chamber equipped with a 8002EVS flat fan nozzle that dispensed at 200 L ha^−1^ and at a pressure of 200 kPa at a height of 50 cm above the plant canopy. The experiments were organized in a fully randomized design, with five plants per plot and five repetitions per treatment for each population and dose, repeating them twice. Twenty-one days after treatment (DAT), plant survival rate of each population was recorded and presented as a percentage relative to the untreated control.

### 2.3. Quantification of Accumulated Shikimic Acid

Considering that glyphosate causes the accumulation of shikimic acid [21], this parameter was evaluated to confirm differences in the level of glyphosate susceptibility between *S. laxum* populations. Samples (~50 mg) of leaf tissue samples were collected from R or S plants and placed into 2 mL tubes that contained 1 mL of a solution composed of 1 M NH_4_H_2_PO_4_ (pH 4.4), prepared with glyphosate concentrations of 0, 0.1, 0.2, 0.4, 0.6, 0.8, or 1 mM. Subsequently, the tubes were incubated for 24 h in a BOD at 25 °C and under light intensity of 850 μmol m^−2^ s^−1^. After, tubes were thawed for 30 min at 60 °C. Then, 250 µL of a solution containing 1.25 NHCl was added into each tube, followed by another incubated period of 15 min at 60 °C. Aliquots of 125 μL were transferred to new 2 mL tubes, where 500 μL of a solution composed of periodic acid and sodium meta periodate (each at a concentration of 0.25% (wt/v)) was added. The new tubes were incubated for 90 min at room temperature (~25 °C). Following this incubation, 500 μL of a solution containing 0.6 N sodium hydroxide and 0.22 M sodium sulfite was added to each tube. The total content of the tubes was transferred to spectrophotometric cuvettes, and the absorbance of the samples was measured at 380 nm. For each *S. laxum* population, three samples were tested at each glyphosate concentration, and experiments were repeated twice.

### 2.4. Determination of Absorption and Translocation Rates with ^14^C-Radilabeled Glyphosate

Differences in the amount of herbicide absorbed and/or translocated may explain resistance to herbicides. Thus, to evaluate these parameters in the *S. alxum* populations, R and S plants were first treated with 500 g ae ha^−1^ glyphosate. Prior to the herbicide application, an aluminum layer was placed over the second most recently expanded leaf. The aluminum layer was removed 30 min later, and 1 µL of radiolabeled solution, composed of commercial glyphosate and radiolabeled glyphosate with ^14^C (glycine-2-^14^C), was applied to the upper surface of this leaf. The radiolabeled solution contained 2.9 mg mL^−1^ glyphosate and 834 Bq µL^−1^ of specific activity. Treated plants were maintained under the controlled conditions described above in a growth chamber until evaluation.

The treated leaves were subjected to a thorough washing process with 50% acetone (1 mL for each wash) at intervals of 24, 48, and 96 h after treatment (HAT) to recover the unabsorbed ^14^C-herbicide. The rinse solution from each wash was collected individually in scintillation vials. Next, 2 mL of liquid scintillation cocktail (Ultima Gold, Perkin-Elmer, BV BioScience Packard, Waltham, MA, USA) was added to each vial. The radioactivity of the samples was quantified as disintegrations per minute (dpm) in a liquid scintillation counter, with each sample undergoing a 10 min measurement period. Plants were carefully uprooted from the pots to assess the translocation rate of ^14^C-glyphosate. Roots were thoroughly cleansed with distilled water, dried using paper towels, and five plants were subsequently divided into treated leaves, other aerial parts of the plant, and the root system. Each plant segment was enclosed in a cellulose cone and subjected to a drying process at 60 °C for 4 days.

After drying, samples were incinerated utilizing an automated oxidation system (Packard Tri Carb 307). During combustion, the system captured the ^14^CO_2_ released and transferred it into an 18 mL mixture comprising a radioactive dioxide absorbent and a liquid scintillation cocktail (Carbo-Sorb E and Permafluor, respectively, at 1:1 (*v*/*v*), Perkin-Elmer, Packard Bioscience BV, Waltham, MA, USA). Radioactivity (in dpm) of the samples was also quantified using a liquid scintillation counter for 10 min. The rates of absorption and translocation of ^14^C-glyphosate were represented as a percentage of the total ^14^C-herbicide applied and absorbed, respectively.

An additional set of three plants from each *S. laxum* population utilized to observe the translocation of ^14^C-glyphosate at 96 HAT. Entire plants were rinsed, fixed on filter paper, and allowed to dry at room temperature (~25 °C) for 10 d. The plants were then placed next to a phosphor storage film (Perkin-Elmer) in the dark for 4 h. The ^14^C-glyphosate translocation was revealed in a phosphor imager Cyclone (Perkin-Elmer).

### 2.5. Quantification of Glyphosate and Potential Metabolites Using CE

Some weed species have the ability to metabolize glyphosate as a resistance mechanism [22]. To evaluate this mechanism, 10 plants of each population of *S. laxum* received a treatment of 500 g ae ha^−1^ glyphosate, while 10 untreated plants served as controls. The aerial part of the plants were cut at ground level and underwent a thorough washing process with distilled water to remove traces of the herbicide on the surface at 96 HAT. Excess water was removed with paper towels, and the samples were immediately frozen in liquid N_2_ to later be stored at 80 °C until used for analysis.

The extraction of glyphosate and its metabolites, amino methyl phosphonic acid (AMPA), glyoxylate, and sarcosine, was performed according to Ref. [22]. For that, samples of 1.5 g of plant tissues were grinded in a mortar for 5 min continuously adding liquid nitrogen. The resulting fine powder was transferred to a plastic beaker and extracted three times with 8 mL water–acetone (1:1 *v*/*v*). Each extraction cycle consisted of magnetic stirring for 10 min, ultrasonication for 5 min, and centrifugation at 41 °C and 10,000 rpm for 15 min [22]. The supernatants from the three extraction cycles were recovered, combined, and evaporated to dryness under a flow of nitrogen. The extract was reconstituted in 2 mL of background electrolyte (10 mM potassium phthalate, 0.5 mM cetrimonium bromide (CTAB), and 10% acetonitrile at pH 7.5) and filtered with nylon filters (45 mm pore size × 13 mm id, Millipore) into bottom sterile polystyrene tubes.

The filtered extract was injected in a 3-D Capillary Electrophoresis Agilent G1600A Instrument, equipped with a DAD (range 190–600 nm), a capillary tubing of 88.5 cm (effective length 80 cm) 50 mm id × 375 mm od (Análisis Vínicos, Ciudad Real, Spain), and thermostated by a Peltier unit. The analysis voltage was −20 kV, and the monitoring wavelength was 220 nm for all analytes. Calibration curves were created by utilizing standard patterns obtained from Sigma-Aldrich (St. Louis, MI, USA). The experimental design of the study was completely randomized, and the data were expressed as percentages relative to the total sum of glyphosate and recovered metabolites.

### 2.6. Enzymatic Interaction of Glyphosate with Its Target Site

To evaluate the insensitivity or overproduction of the glyphosate target site, the EPSPS of R and S plants of *S. laxum* was extracted. Leaf tissue samples (5 g) were collected from S and R *S. laxum* plants, and the extraction of the enzyme was carried out according to Ref. [23]. The quantification of total soluble protein (TSP) associated with EPSPS was ascertained employing a Protein Determination Kit (Sigma-Aldrich, Madrid, Spain), adhering to the manufacturer’s instructions. To evaluate specific EPSPS activity, the commercial kit EnzChek Phosphate Assay (Invitrogen, Carlsbad, CA, USA) was employed by adding glyphosate (with a purity of <99%, Sigma-Aldrich) at varying concentrations spanning from 0 to 1000 µM. The assessment of EPSPS activity involved quantifying the release of inorganic phosphate (Pi) per microgram of TSP per min (µmol Pi µg^−1^ TSP min^−1^). The data of EPSPS activity were expressed as a percentage in relation to the control, which denoted the absence of glyphosate. Three samples from each population were analyzed using the determined glyphosate concentration.

### 2.7. EPSPS Gene Sequencing

Partial sequencing of the conserved region of the *EPSPS* gene was performed to identify possible mutations that confer resistance to glyphosate. Leaf samples of approximately 100 mg were collected from 10 plants of the S and R *S. laxum* population. Samples were immediately placed in 2 mL tubes, flash-frozen in liquid N_2_, and stored at −80 °C. The RNA extraction was performed following the TRIzol purification protocol [24]. To eliminate any DNA contamination, the RNA was treated with TURBO DNase (RNase-Free; Ambion, Warrington, UK) and subsequently stored at −80 °C. The integrity of the RNA was assessed using electrophoresis on a 0.8% agarose gel, and the concentration was determined with a NanoDrop ND-1000. Subsequently, 1 μg of RNA per sample was used to synthesize cDNA, employing an iScript cDNA Synthesis Kit from Bio-Rad Laboratories, Inc. (CA, United States), following the manufacturer’s instructions. PCR reactions (25 µL total volume) were carried out using primers BpF13 and BpR11 (5′-TTGCCYGGRTCMAAGTCTTT-3′ and 5′-GTCCCAASTATCACTRTGTTC-3′, respectively) [25], to amplify a fragment of 639 bp.

Each PCR reaction contained 50 ng of cDNA, 0.2 µM of each primer, 0.2 mM dNTP mix, 2 mM MgCl2, 1X buffer, and 0.625 units of polymerase (*Thermus thermophilus)* and *Pyrococcus furiosus* at 100:1). PCR conditions involved an initial denaturation at 94 °C, followed by 35 cycles of denaturation (94 °C for 30 s), annealing (55 °C for 30 s), and extension (72 °C for 1 min), and a final extension at 72 °C. The quality of PCR products was checked with 1% agarose gel, and then, they were ligated into the pGEM-T Easy Vector System and cloned into *E. coli* DH5α competent cells. Positive transformants were selected and confirmed with PCR using the universal primers M13F (5′-CGCCAGGGTTTTCCCAGTCACGAC-3′) and M13R (5′-TCACACAGGAAACAGCTATGAC-3′) [21]. Plasmids were purified and sequenced using Sanger sequencing. Sequence assembly was conducted using SeqMan Pro (Version 11.0) and Geneious software (Version8.1.8) [25]. Each cDNA sample was amplified in triplicate.

### 2.8. Statistics

The LD_50_ and I_50_ values, which represent the glyphosate concentrations that caused a 50% decrease in the mortality of plants and/or the activity of the EPSPS, respectively, were determined with nonlinear regression analysis using the percentage data from the dose–response curves. The analysis employed the formula *y* = [d/1 + (*x*/*g*)*b*] [26], where *y* is the parameter under consideration relative to the untreated control, *d* corresponds to the upper limit, *b* is the slope of the curve, *g* corresponds to the LD_50_ or I_50_ (inflection point of the curve at the midpoint), and x corresponds to the tested glyphosate concentration analyzed as an independent variable. The resistance factors (RF) for each parameter were calculated as R-to-S ratio (RF = R/S).

Pairwise Student’s *t*-tests were conducted to compare the data of shikimic acid, ^14^C-glyphosate absorption and translocation, and EPSPS basal activity between the S and R *S. laxum* populations, using the software Statistix 9 (Analytical Software, Mckinney, TX, USA). Significance was established at *p* ≤ 0.05.

## 3. Results

### 3.1. Confirmation of Resistance

**Figure 2:**
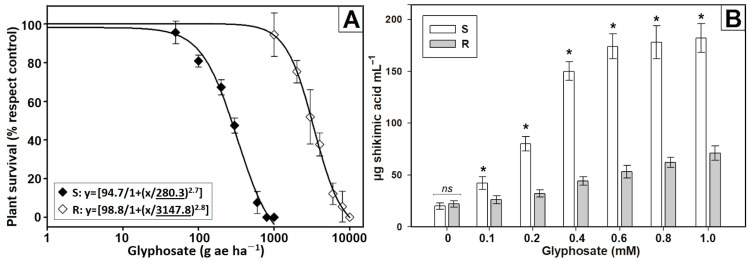
(**A**) Dose–response curves of *Steinchisma laxum* populations (S, susceptible and R, resistant) treated with varying doses of glyphosate. The vertical bars in the graph show the standard error (n = 20). The digits underlined in the nonlinear regression equation correspond to the parameter ‘g’, representing the LD_50_ values. (**B**) Shikimic acid accumulation in *S. laxum* plants at various glyphosate concentrations. The vertical bars in the graph show the standard error (n = 6). The asterisk (*) is used to indicate statistical significance as determined using the pairwise Student’s *t*-test (*p* < 0.05), whereas ‘ns’ denotes the absence of a significant differences.

In dose–response experiments, each population of *S. laxum* exhibited different levels of susceptibility to glyphosate (Figure 2A). The estimated LD_50_ for the R population was 3147 g ae ha^−1^ (95%CI 2888–3407), while that of the S population was only 280 g ae ha^−1^ (95%CI 253–307). These values indicate that the R population displayed a remarkable 11.2-fold (FR) greater resistance to glyphosate compared to the S population. In the shikimic acid accumulation experiments, the controls of S and R *S. laxum* populations displayed similar basal shikimic acid content (~21 µg shikimate mL^−1^) in absence of glyphosate. Although both populations accumulated shikimic acid in the presence of glyphosate, the accumulation was consistently higher in the S population. This population achieved its maximum accumulation (174 µg shikimate mL^−1^) from 0.6 mM glyphosate, while the accumulation of the R population increased gradually as the glyphosate concentrations increased, reaching 71 µg shikimate mL^−1^ at 1 mM glyphosate (Figure 2B).

### 3.2. Characterization of Non-Target Site Mechanisms

**Figure 3:**
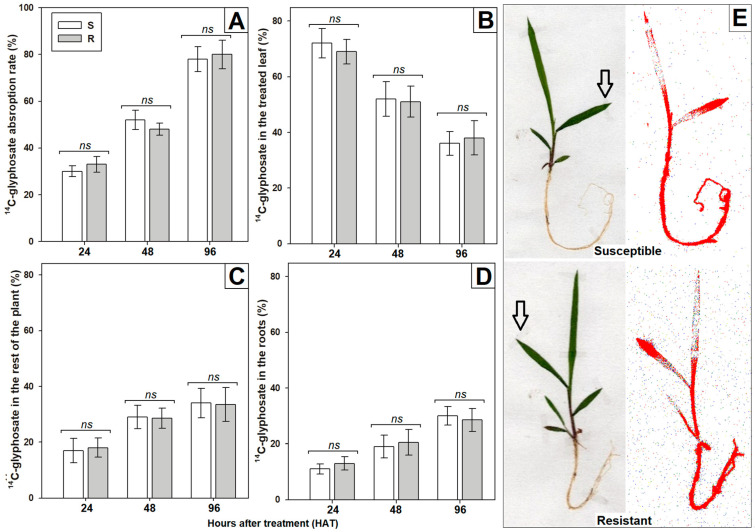
Percentages of ^14^C-glyphosate absorption (**A**) (calculated from % recovered) and translocation (calculated from % absorbed) from the treated leaf (**B**) to other parts of the plant (**C**) and root system (**D**) in *Steinchisma laxum* populations (S, susceptible and R, resistant) at 24, 48, and 96 h after treatment (HAT). The vertical bars in the graphs show the standard error (n = 5), and ‘ns’ denotes the absence of a significant difference using the pairwise Student’s *t*-test (*p* < 0.05). (**E**) Images captured digitally and through autoradiography depicting the ^14^C-glyphosate distribution within both S and R *S. laxum* plants at 96 HAT. The red zones indicate areas with the highest concentration of ^14^C-glyphosate. Arrows highlight the treated leaf.

The absorption and translocation rates of ^14^C-glyphosate did not differ between S and R *S. laxum* populations at all evaluated time intervals. The absorption ranged from 30–33% at 24 HAT to nearly 80% at 96 HAT (Figure 3A). Both populations translocated substantial amounts of ^14^C-herbicide. At 24 HAT, the majority of absorbed ^14^C-glyphosate (70%) was still located in the treated leaf (Figure 3B), but at 96 HAT, approximately 34% had been transported to other parts of the plant and 29% reached the root system (Figure 3C,D).

None of the *S. laxum* populations were able to metabolize glyphosate. Over 96% of the herbicide was found in its chemically pure form, with only a small percentage of AMPA detected in R and S plants. The other potential glyphosate metabolites, glyoxylate and sarcosine, were not detected (Table 1).

### 3.3. Characterization of Target Site Mechanisms

**Figure 4:**
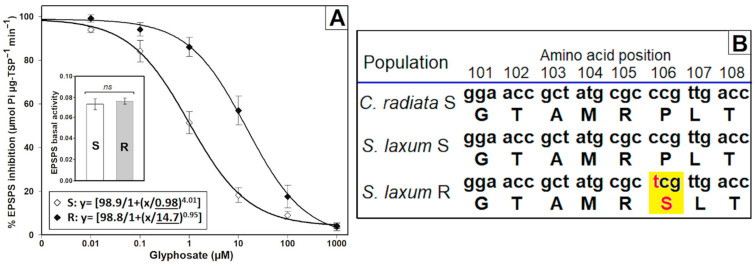
(**A**) Enzyme activity and sequencing of the 5-enolpyruvilshikimato-3-phosphate synthase (EPSPS) in *Steinchisma laxum* populations (S, susceptible and R, resistant). (**A**) Basal activity and inhibition rate of the EPSPS with glyphosate. The vertical bars in the graphs show the standard error (n = 5), and ‘ns’ denotes the absence of a significant difference using the pairwise Student’s *t*-test (*p* < 0.05). The digits underlined in the nonlinear regression equation correspond to the parameter ‘g’, representing the I_50_ values. (**B**) Partial alignment of the predicted amino acids derived from the gene responsible for encoding *EPSPS* in *S. laxum* populations compared to the susceptible *Chloris radiata* population (ChrS) from Colombia [11]. Yellow box highlights substitution of nucleotides and amino acids at position 106 of the gene encoding *EPSPS* in *Arabidopsis thaliana* (GenBank: CAA29828.1), using the start codon (ATG) as a reference.

In the enzyme activity assays, no differences were found in the basal activity (without glyphosate) of the EPSPS between the *S. laxum* populations. The amount of EPSPS quantified in the absence of glyphosate was 0.073 and 0.077 μmol Pi μg^−1^ TSP min^−1^ in the S and R plants, respectively. However, the enzymatic activity of the EPSPS was inhibited to different extents in each population. The EPSPS of the R population required 15 times more glyphosate to be inhibited by 50% compared to the S population (I_50_ = 0.98 µM) (Figure 4A). The analysis of the gene encoding *EPSPS* uncovered a cytosine (C) nucleotide substitution to thymine (T) in the first position of the codon that codes for amino acid 106. This alteration led to the replacement of serine (S) with proline (P) (Figure 4B).

## 4. Discussion

The dose of glyphosate required for a significant lethal effect on the R *S. laxum* population was much higher (11.2 times) relative to S population. The elevated LD_50_ value indicates that R individuals exhibit lower susceptibility to the herbicide [27,28], suggesting an evolution of glyphosate resistance. In addition, the high LD_50_ value of the R population was well above the field dose (1080 g ae ha^−1^). This resistance verdict was further corroborated by the results of the shikimic acid quantification experiments, where the R population accumulated substantially less shikimate than the S population. Glyphosate hinders the activity of EPSPS in susceptible plants, causing the accumulation of shikimic acid, which impairs amino acid synthesis and ultimately leads to plant death [21,29]. The low accumulation of shikimate in glyphosate-resistant plants may stem from genetic adaptations within the population regulating resistance mechanisms that diminish the sensitivity/interaction of EPSPS with glyphosate [16,30]. Consequently, resistant plants can sustain essential amino acid synthesis and survive glyphosate treatment.

^14^C-glyphosate was absorbed in the treated leaves and translocated to other parts of the plant and root system equally in S and R *S. laxum* plants, i.e., no physiological mechanism prevents glyphosate from reaching its target site [31,32]. Additionally, there was an absence of glyphosate metabolism, which explained the lack of herbicide metabolites in both populations [33,34]. This suggests that the non-target site type mechanisms characterized in this study played no role in the resistance to glyphosate of the R *S. laxum* population; however, this does not exclude the existence of other resistance mechanisms in the resistant plants.

Glyphosate resistance can arise due to molecular mechanisms, such as overexpression or mutations in the *EPSPS* gene [35]. Since the basal activity of both *S. laxum* populations was similar, resistance to glyphosate cannot be linked to EPSPS overexpression [36]. However, the divergent EPSPS inhibition rate by glyphosate between *S. laxum* population suggested the potential occurrence of a mutation in the gene encoding *EPSPS* of the R population. The analysis of the gene encoding *EPSPS* of this population confirmed the presence of the mutation Pro106Ser. Mutations at this position modify the structural and functional conformation of EPSPS [37,38]. The Pro substitution by Ser at 106 position decreases the binding of glyphosate to the enzyme, diminishing the interaction of the active site of the EPSPS with the herbicide [39]. In addition to the Pro to Ser substitution, additional alterations in amino acids have been documented at the Pro106 position, including Ala, Gly, His, Leu, and Thr [39,40,41]. As a result, the mutated EPSPS gene at the Pro-106 position becomes less sensitive to glyphosate, allowing the EPSPS to maintain proper functionality in the route of shikimic acid in R plants when the herbicide is present. The selection pressure with glyphosate should be reduced in the R *S. laxum* population that presents the mutation Pro106Ser, and in other putative resistant populations, there is a great risk of double (TIPS and TIPT) and triple (TAP-IVS) mutations [25,42,43,44], which increase the degree of glyphosate resistance, being present in these species.

Because mutations at 106 position are often reported as conferring low levels glyphosate resistance (2–4 fold) [35,37,39], the high level of resistance of 11-fold observed in the R population of *S. laxum* could be questioned. However, biotypes of *Conyza canadensis* from Ohio and Iowa, USA, presented high glyphosate resistance levels, with survival rates greater than 80% at doses of 20- or 40-fold the field dose (840 g ae ha^−1^) [45]. The authors mainly attribute this extreme resistance mainly to the Pro106Ser mutation, although they did not rule out the synergistic action with the NTSR mechanism [45]. Because the other mechanisms (TRS and NTSR) characterized in this research do not contribute to glyphosate resistance of the R *S. laxum* population, it is possible that mechanisms that were not studied, such as cell exclusion [46], or have not yet been described, could be implicated in the glyphosate resistance observed in this population.

## 5. Conclusions

This study findings demonstrate a significant difference in the glyphosate susceptibility between the R and S populations of *S. laxum* occurring in rice fields from Colombia. The presence of the mutation Pro106Ser in the gene encoding *EPSPS* explains partially the observed glyphosate resistance by affecting the structural and functional conformation of the enzyme, leading to reduced affinity for glyphosate. The comparable absorption, translocation, and metabolism patterns in both populations showed that non-target site resistance mechanisms played no role in the resistance to glyphosate in the R population. However, other unstudied resistance mechanisms may also contribute to resistance.

## Figures and Tables

**Table 1:** Glyphosate metabolism in *Steinchisma laxum* plants (S = susceptible and R = resistant) subjected to a glyphosate application at a rate of 360 g ae ha^−1^ at 96 h after treatment.

Compound	Population	
	S	R
Glyphosate ^ns^	96.2 ± 2.7	97.4 ± 3.6
AMPA ^ns^	3.8 ± 2.2	2.6 ± 1.8
Glyoxylate	nd	nd
Sarcosine	nd	nd

AMPA: amino methyl phosphonic acid. ‘nd’ indicates non-detection. ± are the standard error (n = 5), and ‘ns’ denote the absence of a significant difference using the pairwise Student’s *t*-test (*p* < 0.05).

## Data Availability

Raw data will be shared as requested.
